# Adverse prognosis associated with asymmetric myocardial thickening in aortic stenosis

**DOI:** 10.1093/ehjci/jex052

**Published:** 2017-03-30

**Authors:** Jacek Kwiecinski, Calvin W L Chin, Russell J Everett, Audrey C White, Scott Semple, Emily Yeung, William J Jenkins, Anoop S V Shah, Maria Koo, Saeed Mirsadraee, Chim C Lang, Nicholas Mills, Sanjay K Prasad, Maurits A Jansen, Alan G Japp, David E Newby, Marc R Dweck

**Affiliations:** 1 BHF/Centre for Cardiovascular Science, University of Edinburgh, 49 Little France Crescent, Edinburgh EH16 4SB, UK; 2 First Department of Cardiology, Poznan University of Medical Sciences, 1/2 Dluga, 61-848 Poznan, Poland; 3 Department of Cardiovascular Science, National Heart Center Singapore; 4 Clinical Research Imaging Centre, University of Edinburgh, UK; 5 Division of Cardiovascular and Diabetes Medicine, Ninewells Hospital and Medical School, UK; 6 Royal Brompton Hospital, London, UK; 7 Translational and Molecular Imaging Institute, Icahn School of Medicine at Mount Sinai, New York

**Keywords:** aortic stenosis, asymmetric wall thickening, magnetic resonance, echocardiography, hypertrophy, troponin

## Abstract

**Aims:**

Asymmetric wall thickening has been described in patients with aortic stenosis. However, it remains poorly characterized and its prognostic implications are unclear. We hypothesized this pattern of adaptation is associated with advanced remodelling, left ventricular decompenzation, and a poor prognosis.

**Methods and results:**

In a prospective observational cohort study, 166 patients with aortic stenosis (age 69, 69% males, mean aortic valve area 1.0 ± 0.4 cm^2^) and 37 age and sex-matched healthy volunteers underwent phenotypic characterization with comprehensive clinical, imaging, and biomarker evaluation. Asymmetric wall thickening on both echocardiography and cardiovascular magnetic resonance was defined as regional wall thickening ≥ 13 mm and > 1.5-fold the thickness of the opposing myocardial segment. Although no control subject had asymmetric wall thickening, it was observed in 26% (*n* = 43) of patients with aortic stenosis using magnetic resonance and 17% (*n* = 29) using echocardiography. Despite similar demographics, co-morbidities, valve narrowing, myocardial hypertrophy, and fibrosis, patients with asymmetric wall thickening had increased cardiac troponin I and brain natriuretic peptide concentrations (both *P* < 0.001). Over 28 [22, 33] months of follow-up, asymmetric wall thickening was an independent predictor of aortic valve replacement (AVR) or death whether detected by magnetic resonance [hazard ratio (HR) = 2.15; 95% confidence interval (CI) 1.29–3.59; *P* = 0.003] or echocardiography (HR = 1.79; 95% CI 1.08–3.69; *P* = 0.021).

**Conclusion:**

Asymmetric wall thickening is common in aortic stenosis and is associated with increased myocardial injury, left ventricular decompenzation, and adverse events. Its presence may help identify patients likely to proceed quickly towards AVR.

**Clinical Trial Registration:**

https://clinicaltrials.gov/show/NCT01755936: NCT01755936.

## Introduction

Aortic stenosis is not only characterized by progressive valve narrowing but also by the hypertrophic response of the left ventricle (LV) that ensues.^1^ Indeed the development of symptoms and adverse events appears as much related to events in the myocardium as the valve.^2^ New techniques for assessing adverse patterns of remodelling are therefore of major interest.^3^

Echocardiography is the most common imaging technique to assess patients with aortic stenosis and can provide assessments of wall thickness that can be used to calculate left ventricular mass index. Cardiovascular magnetic resonance is less widely employed but provides the gold standard assessment of left ventricular mass and wall thickness with the unique ability to identify myocardial fibrosis. Asymmetric wall thickening is most commonly associated with hypertrophic cardiomyopathy.^4^ However this form of remodelling has recently been described in patients with increased afterload such as hypertension and aortic stenosis.^5–8^ An initial magnetic resonance study suggested that asymmetric wall thickening could be observed in around a quarter of patients. However, this study was hampered by referral bias and did not involve detailed tissue characterization.^6^ Echocardiographic studies have suggested a lower prevalence of around 10%, with the prognostic implications of this observation remaining unclear.^7^^,^^8^

In this study, we sought to assess asymmetric wall thickening in patients with aortic stenosis using both cardiovascular magnetic resonance and echocardiography. In particular, we aimed to investigate in depth the factors associated with an asymmetric pattern of wall thickening, its relationship with other markers of left ventricular remodelling and decompenzation and to assess its prognostic implications.

## Methods

### Patient population

We performed a prospective observational cohort study of stable subjects with mild, moderate, and severe aortic stenosis recruited from the Edinburgh Heart Centre. All patients who attended the institution between March 2012 and August 2014 were invited to participate. We excluded patients with other forms of valvular heart disease (moderate or severe in nature), end-stage heart failure, advanced malignancies or other comorbidities with a life expectancy < 2 years, cardiomyopathies (including previous myocarditis), and contraindications to gadolinium-enhanced magnetic resonance, such as ferromagnetic foreign bodies and estimated glomerular filtration rate < 30 mL/min/1.73 m^2^. Coronary artery disease was defined as previous myocardial infarction, documented evidence of myocardial ischaemia or a > 50% stenosis of the coronary artery lumen. The blood sampling and analysis protocols are described in the Supplementary data online.

Both the clinical and imaging assessments were carried out at the Clinical Research Facility and the Clinical Research Imaging Centre, Edinburgh. The study was conducted in accordance with the declaration of Helsinki and approved by the local research committee. Written informed consent was obtained from all enrolled patients.

### Cardiovascular magnetic resonance

Cardiovascular magnetic resonance was performed using a 3T scanner (MAGNETOM Verio, Siemens Healthcare GmbH, Erlangen, Germany). A balanced steady-state free precession sequence was used for short-axis cine imagine of the LV and assessment of left ventricular volumes, mass and ejection fraction (Argus software, Siemens AG, Healthcare Sector). LV longitudinal function was determined by measuring the difference in mitral annular displacement between end-systole and end-diastole. On short-axis cine images, epicardial and endocardial contours were carefully identified and planimetered in end-systole and end-diastole for left ventricular volume quantification. The left ventricular mass was calculated from the total myocardial volume (excluding trabeculations and papillary muscles) multiplied by the density of the myocardium (1.05 g/mL). All volumes and mass values were indexed to body surface area (calculated using the Du Bois formula). The left ventricular mass/volume ratio (M/V) was calculated by dividing the left ventricular mass by the left ventricular end-diastolic volume. This parameter indexes the left ventricular mass to cavity size, with M/V values > 1.16 g/mL identifying patients with a relative increase in wall thickness.^6^ Left ventricular hypertrophy was defined as an indexed left ventricular mass ≥ 95th centile of the normal range corrected for age and gender.^9^ Maximal wall thickness was evaluated in all 17 segments of the LV from cine images of the LV in end-diastole (again excluding ventricular trabeculations). Asymmetric left ventricular wall thickening was defined as a regional wall thickening ≥ 13 mm that was also ≥ 1.5-fold the thickness of the opposing myocardial segment.^6^ Such criteria had to be fulfilled on two adjacent short-axis magnetic resonance cine images.

### Fibrosis assessment

Late gadolinium enhancement (LGE) was used for detection of focal replacement fibrosis. Acquisition was performed between 8 and 15 min after gadobutrol (Gadovist, Bayer Pharma AG, Germany) administration using an inversion recovery fast gradient-echo sequence and a phase-sensitive inversion recovery sequence in two phase-encoding directions in order to distinguish true enhancement from artefact. The inversion time was optimized to achieve adequate nulling of the myocardium. The presence of LGE was evaluated visually by two experienced operators (Marc R. Dweck & Calvin W.L. Chin.). Where present, it was quantified using QMASS software (Medis, Leiden, The Netherlands) as an area of myocardium with a signal intensity exceeding the threshold of two standard deviations above the mean value of normal myocardium. All areas of inversion artefact or myocardial regions contaminated by blood pool or epicardial fat were excluded.

T1 mapping was performed using the Modified Look-Locker Inversion recovery sequence for the assessment of diffuse myocardial fibrosis. Short-axis T1 maps of the mid-cavity were acquired in diastole before and 20 min after the administration of 0.1 mmol/kg of gadobutrol (Gadovist, Bayer Pharma AG, Germany) using a dedicated 32-channel anterior-posterior cardiac array. T1 maps were analysed using OsiriX 4.1.1 software (Geneva, Switzerland). For diffuse fibrosis, we calculated the extracellular volume fraction (ECV) derived from pre- and post-contrast myocardial T1 values corrected for blood-pool T1 and haematocrit. The ECV was calculated according to: ECV = partition coefficient ×  [1 − haematocrit], where partition coefficient = [ΔR1_myocardium_/ΔR1_blood-pool_] and ΔR1 = (1/post-contrast T1 − 1/pre-contrast T1). In order to evaluate the total amount of interstitial fibrosis in our study cohort, we calculated the indexed fibrosis volume in each patient using the following equation: ECV × left ventricular myocardial volume corrected for the body surface area.

### Echocardiography

All participants underwent a transthoracic echocardiographic examination for the assessment of aortic stenosis and cardiac function on iE33, Philips Medical Systems, The Netherlands. The severity of aortic stenosis was classified on the basis of the aortic jet peak velocity, the mean pressure gradient and the aortic valve area derived using the continuity equation. All assessments were conducted in accordance with European Association of Echocardiography/American Society of Echocardiography (ASE) guidelines.^10^ Transmitral early (E) and late diastolic velocities and deceleration time were measured at the tips of mitral valve leaflets using pulse wave Doppler. Pulse-wave tissue Doppler imaging was used to evaluate the early (e’) diastolic velocities of the medial and lateral mitral annulus. Diastolic function was determined using the E/e’ ratio. The left ventricular mass was calculated using wall thickness measurements and cavity dimensions (ASE formula) and indexed to body surface area.^11^ Cut-off values of 115 g/m^2^ for males and 95 g/m^2^ for females were used to distinguish subjects with left ventricular hypertrophy. Relative wall thickness (RWT) calculated according to the formula: RWT = 2PWTd/LVEDD (PWTd posterior wall thickness at end-diastole; LVEDD LV end-diastolic dimension) was used in a similar fashion to M/V to classify subjects into the different patterns of remodelling and hypertrophy (see Supplementary data online, *Table S1*). To assess the presence of asymmetric wall thickening, both long and short-axis images were screened by two experienced operators blinded to the magnetic resonance data (A.G.J. and J.K.). Similar to magnetic resonance assessments, asymmetric left ventricular wall thickening was defined as a regional wall thickening ≥ 13 mm that was also ≥ 1.5-fold the thickness of the opposing myocardial segment.

### Patterns of left ventricular adaptation

Using both echocardiography and Cardiovascular Magnetic Resonance (CMR), we categorized patients with aortic stenosis into six groups of anatomic adaptation based on the left ventricular mass index, the indexed left ventricular end-diastolic volume, M/V and the presence of asymmetric wall thickening.^6^*Normal* ventricular structure, c*oncentric remodelling*, a*symmetric remodelling, concentric hypertrophy*, a*symmetric hypertrophy*, *and eccentric hypertrophy* (see Supplementary data online, *Table S1*).

### Clinical endpoints

The primary endpoint of the study was aortic valve replacement and all-cause mortality. In the subgroup of subjects that subsequently underwent aortic valve replacement, we determined the impact of asymmetric thickening on 30-day perioperative cardiovascular outcomes (myocardial infarctions, congestive heart failure, new episodes of atrial or ventricular arrhythmia, perivalvular leaks, permanent pacemaker insertion, cardiac tamponade). Patients that underwent AVR were censored for survival analysis and considered as withdrawn alive. All the mortality, surgery, and in-hospital complications data were obtained from the National Strategic Tracing Service, which is a national database for all National Health Service patients in UK.

### Statistical analysis

We assessed the distribution of all continuous variables using the Shapiro–Wilk test, and presented them as mean ± standard deviation or median (interquartile range). Comparisons were made using one-way analysis-of-variance to compare continuous parametric data and the Kruskall–Wallis test for non-parametric data. Chi-square tests were used for categorical baseline characteristics. The association between biomarkers and asymmetric wall thickening was assessed using linear regression analyses with adjusting for potential confounders. Kaplan–Meier curves were used to elucidate the survival distributions with regard to all-cause mortality and AVR. Differences in the outcome of patients with and without asymmetric wall thickening were assessed using the log-rank test. A Cox proportional hazard regression with adjustment for potential confounders was performed to determine the predictors of worse outcome. A two-sided *P* < 0.05 was considered statistically significant. All statistical analysis has been performed using SPSS Version 20 (IBM Corp., Armonk, NY, USA) and GraphPad Prism Version 5.0 (GraphPad Software, San Diego, CA, USA).

## Results

### Study population

The study group comprised 166 patients with aortic stenosis (69 [63,75] years old, 68% males) and 37 age- and sex-matched healthy volunteers (68 [63,74] years; 65% males). A comprehensive overview of patients’ demographics, aortic stenosis severity, left ventricular characteristics, and co-morbidities as well as comparisons to healthy controls can be found in *Table 1* and Supplementary data online.

**Table 1:** Comparison of patient characteristics between those with concentric wall thickening and asymmetric wall thickening on magnetic resonance

	Concentric wall thickening	Asymmetric wall thickening	
	(*n* = 67)	(*n* = 43)	*P*-value
Baseline characteristics			
Age, years	70 [65,77]	72 [67,75]	0.56
Males, *n* (%)	52 (77)	31 (72)	0.52
CAD, *n* (%)	22 (33)	20 (47)	0.16
Diabetes, *n* (%)	9 (13)	7 (16)	0.80
Hyperlipidaemia, *n* (%)	38 (57)	22 (51)	0.84
Hypertension, *n* (%)	48 (72)	33 (77)	0.56
SBP, mmHg	150 ± 20	153 ± 22	0.53
Six minute walk, distance (m)	369 ± 96	358 ± 124	0.64
Symptomatic AS, *n* (%)	16 (24)	14 (32)	0.31
Echocardiography			
AVA, cm^2^	0.8 [0.7,1.1]	0.8 [0.7,1.0]	0.15
AVA indexed, cm^2^/m^2^	0.44 [0.38,0.58]	0.43 [0.36,0.50]	0.08
Dimensionless index	0.25 [0.21,0.30]	0.23 [0.19,0.28]	0.06
Vm, m/s	3.9 [3.4,4.5]	4.2 [3.8,4.8]	0.01
MPG, mmHg	35 [24,44]	41 [34,50]	0.01
Mild, *n* (%)	10 (15)	1 (2)	0.02
Moderate, *n* (%)	18 (27)	9 (22)	0.47
Severe, *n* (%)	39 (58)	33 (76)	0.05
Indexed SV < 35 mL/m^2^, *n* (%)	13 (19)	9 (21)	0.85
E/A	0.81 [0.68, 1.00]	0.82 [0.63, 1.16]	0.68
Deceleration time	206 [169, 254]	217 [196, 247]	0.41
E/e'	12.6 [9.8,16.7]	14.2 [11.5,18.5]	0.05
LVOT Vm, m/s	1.0 [0.9,1.1]	1.0 [0.9,1.2]	0.29
Bicuspid Aortic Valve n (%)	26 (39)	14 (33)	0.51
Cardiovascular magnetic resonance			
Indexed EDV, mL/m^2^	67 [60,74]	68 [62,78]	0.15
Indexed ESV, mL/m^2^	22 [16,26]	22 [18,26]	0.42
Indexed SV, mL/m^2^	46 ± 9	48 ± 9	0.26
Indexed SV < 35 mL/m^2^, *n* (%)	6 (9%)	4 (8%)	0.94
Max wall thickness, mm	12 [10,13]	16 [14,17]	<0.001
Indexed left ventricular mass, g/m^2^	92 [81,103]	96 [80,106]	0.49
Left ventricular mass/EDV, g/mL	1.34 [1.24,1.56]	1.36 [1.21,1.50]	0.39
Mid-wall fibrosis, *n* (%)	25 (37)	21 (48)	0.24
Extracellular volume fraction, %	27.6 ± 2.9	28.1 ± 2.6	0.29
Indexed fibrosis volume, mL/m^2^	24.5 [20.7,29.4]	26.6 [21.1,30.4]	0.28
Ejection fraction, %	68 [64,72]	67 [64,73]	0.92
Longitudinal function, mm	11.8 ± 3.0	11.1 ± 2.6	0.18
Biomarkers			
HS-cTnI, ng/L	6.6 [4.3,10.38]	13.5 [8.1,32.8]	<0.001
BNP, pg/mL	20.9 [8.1,51.8]	56.3 [25.5,112]	<0.001
Outcomes			
Combined primary outcome, n (%)	34 (51)	35 (81)	0.001
AVR, *n* (%)	28 (42)	31 (72)	0.002
Aortic stenosis-related death, *n* (%)	2 (3)	2 (5)	0.78
All cause death, *n* (%)	7 (10)	6 (14)	0.58

CAD, coronary artery disease; SBP, systolic blood pressure; AS, Aortic Stenosis; MPG, mean pressure gradient; LVOT, left ventricular outflow track; EDV, end diastolic volume; ESV, end systolic volume; SV, stroke volume; HS-cTnI, high-sensitivity cardiac troponin I; AVR, aortic valve replacement.

### Cardiovasculur magnetic resonance

#### Patterns of left ventricular adaptation

Using magnetic resonance criteria, 39 patients with aortic stenosis (23%) had normal left ventricular structure. Thirty-four (20%) patients with aortic stenosis had left ventricular remodelling with 22 having a concentric pattern (13%) and 12 (7%) an asymmetric pattern. Among the 93 (58%) patients with left ventricular hypertrophy, the most frequently occurring adaptation pattern was concentric left ventricular hypertrophy detected in 45 individuals (27%), whilst asymmetric left ventricular hypertrophy was observed in 31 subjects (19%). Seventeen (10%) patients with aortic stenosis had an eccentric pattern of hypertrophy, with these patients often having associated aortic regurgitation (*n* = 13), mitral regurgitation (*n* = 2) or a history of myocardial infarction (*n* = 2). All 37 healthy volunteers that comprised the control group had normal left ventricular structure.

#### Asymmetric wall thickening

**Figure 1:**
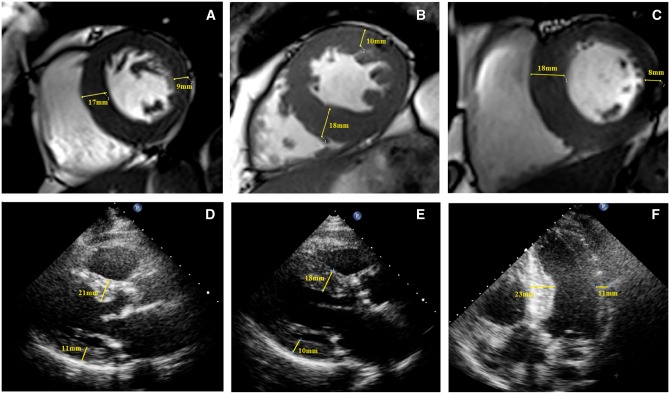
Asymmetrical wall thickening on both magnetic resonance and echocardiography. Images demonstrating asymmetric wall thickening in patients with aortic stenosis. Cardiovascular magnetic resonance short-axis cine images showing an abnormally thickened septum: in a patient with asymmetric remodelling (*A*) and two subjects with asymmetric hypertrophy (*B*) and (*C*). Echocardiographic parasternal long-axis images demonstrating thickening of the septum in two further patients with asymmetric remodelling (*D*) and (*E*). Echocardiographic apical 4-chamber image in a subject with asymmetric hypertrophy (*F*).

**Figure 2:**
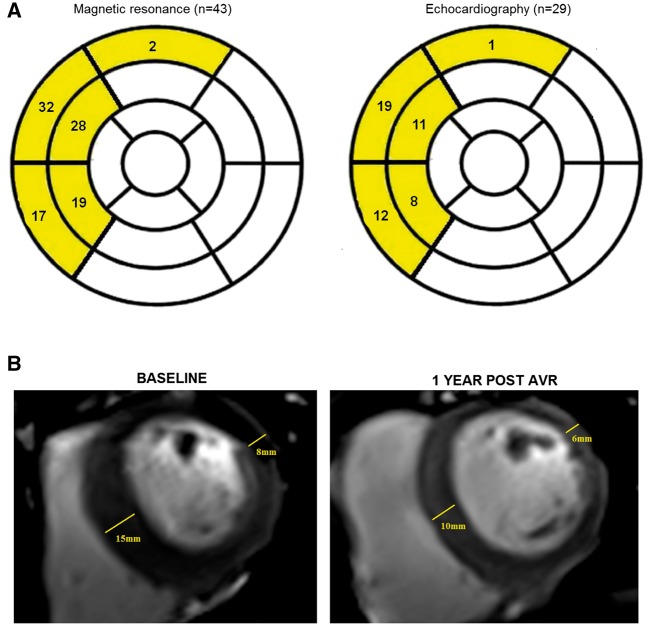
Prevalence, distribution, and resolution after aortic valve replacement of asymmetric wall thickening. (*A*) Seventeen segment model of the LV demonstrating the site of asymmetric wall thickening as detected by both magnetic resonance and echocardiography. Magnetic resonance was more sensitive in detecting asymmetric wall thickening (43 cases) than echocardiography (29 cases). On both modalities, asymmetric wall thickening was almost universally confined to the basal and mid-cavity segments of the septum. (*B*) Patient with asymmetric wall thickening at baseline, which resolved when magnetic resonance was repeated 1 year after aortic valve replacement.

Overall 43 (26%) of our patients with aortic stenosis demonstrated evidence of asymmetric wall thickening on magnetic resonance (*Figure 1*). Twelve patients had asymmetric remodelling and 31 had asymmetric hypertrophy. Importantly none of the healthy volunteers exhibited such a pattern of left ventricular adaptation. The site of asymmetric wall thickening was almost universally in the septum: at the basal level in 33 patients (77% of those with asymmetric wall thickening) and at the mid-cavity in 27 (63%) (*Figure 2A*). In 2 patients (5%), the regional wall thickening was observed in the anterior wall, it was never observed in the lateral or inferior walls. In 13 patients (30%), regional thickening affected just 1 segment of the 17-segment model, 16 subjects (37%) had 2 segments of asymmetric thickening whilst 14 patients (33%) had 3 or 4 affected segments.

**Figure 3:**
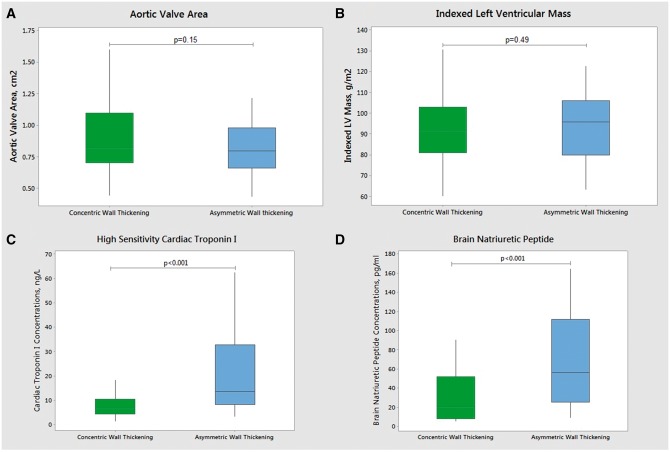
Characteristics of patients with asymmetric vs. concentric wall thickening. Boxplots presenting: aortic valve area (*A*), indexed left ventricular mass (*B*), high sensitivity cardiac troponin I (*C*) and brain natriuretic peptide (*D*) concentrations in aortic stenosis patients with asymmetric and concentric patterns of wall thickening. Despite no difference in AVA and left ventricular mass index (*P* = 0.15 and *P* = 0.49, respectively) patients with asymmetric wall thickening had higher cardiac troponin and BNP levels than those with concentric wall thickening (*P* < 0.001).

We compared patients with asymmetric wall thickening on CMR to patients with concentric patterns of remodelling or hypertrophy (*Table 1*). As expected patients in the asymmetric group had increased maximal wall thickness compared to the concentric groups (16 [14,17] vs. 12 [10,13] mm, *P* < 0.001) but interestingly they also had similar aortic stenosis severity. Indeed, there was no difference between the groups in terms of the aortic valve area (0.8 [0.7,1.1] vs. 0.8 [0.7,1.0] cm^2^, respectively, *P* = 0.15), the indexed aortic valve area (0.43 [0.36,0.50] vs. 0.44 [0.38,0.58] cm^2^/m^2^, *P* = 0.08) nor the dimensionless index (0.25 [0.21,0.30] vs. 0.23 [0.19,0.28], *P* = 0.06). Whilst slightly higher peak aortic jet velocities (4.2 [3.8,4.8] vs. 3.9 [3.4,4.5] m/s, *P* = 0.01) and mean gradients (41([34,50] vs. 35 [24,44], *P* = 0.01) were observed in those with asymmetric wall thickening these differences were small. No differences were observed between the two groups in terms of comorbidities, the magnitude of the hypertrophic response nor the degree of myocardial fibrosis (all *P* > 0.15). Despite these similarities patients with an asymmetric pattern had double the plasma concentrations of troponin I (13.5 [8.1,32.8] vs. 6.6 [4.3,10.4] ng/L, *P* < 0.001) and brain natriuretic peptide (BNP) (56.3 [25.5,112.2] vs. 20.9 [8.1,51.8] pg/mL, *P* < 0.001) compared to subjects with concentric wall thickening. Indeed asymmetric wall thickening was associated with both troponin and BNP levels independent of age, sex, systolic blood pressure, aortic stenosis severity, and left ventricular mass index (*P* < 0.001) (*Figure 3*).

#### Asymmetric wall thickening resolution

Out of the 31 subjects with asymmetric wall thickening who underwent aortic valve replacement, 12 had a repeat magnetic resonance imaging 1-year following surgery. In these patients, the left ventricular mass index decreased on repeat imaging (from 97 [86,102] to 68 [65, 80] g/m^2^; *P* < 0.001) as did maximum wall thickness (from 15 [14,16] to 13 [11,14] mm; *P* = 0.006) with an observed tendency to reduced high-sensitivity troponin I levels (from 9.0 [4.9,20.8] to 4.0 [1.8,10.0] ng/L; *P* = 0.073) (*Figure 2B*). Overall 6 of the 12 patients had complete resolution of their regional wall thickening and no longer fulfilled criteria for asymmetric wall thickening.

#### Fibrosis

Asymmetric wall thickening was associated with higher ECV and replacement fibrosis compared to healthy controls and AS subjects with a normal LV (28.1 ± 2.6% and 48% vs. 26.5 ± 1.3% and 0% and 27.2 ± 2.0 and 14% respectively, all *P* < 0.05). Interestingly there was no significant difference in the fibrosis burden between subjects with an asymmetric and concentric pattern of LV adaptation (28.1 ± 2.6% and 48% vs. 27.6 ± 2.9 and 37%, *P* = 0.29 and *P* = 0.24, respectively).

### Echocardiography

Using echocardiographic criteria, 26 aortic stenosis patients (16%) had normal left ventricular structure. Thirty-two (19%) had left ventricular remodelling whilst 108 (65%) exhibited left ventricular hypertrophy, of whom 9 (6%) had eccentric hypertrophy. Overall echocardiography demonstrated good agreement with magnetic resonance in terms of determining the pattern of left ventricular adaptation (all *P* > 0.10 for a difference) (see Supplementary data online, *Table S4*).

We were interested whether echocardiography was similarly able to detect asymmetric wall thickening. Short-axis images of the LV were either unavailable or non-interpretable in 31 subjects, in whom analysis was based solely on the parasternal and apical long-axis images. Overall 29 of the 166 patients with aortic stenosis exhibited asymmetric wall thickening (17%), again it was not observed in the control patients. Compared to magnetic resonance (which served as the gold standard in this analysis) echocardiography was less sensitive (67%) but showed excellent specificity (100%) in detecting patients with asymmetric wall thickening. Half (*n* = 8, 57%) of the patients with asymmetric wall thickening on magnetic resonance that were missed by echocardiography had non-interpretable short-axis echo images. At the segment level echocardiography missed 47 (48%) of the segments with asymmetric thickening detected by magnetic resonance, although the distribution of this thickening was similar to magnetic resonance: confined to the septum with the exception of 1 patient with anterior wall involvement (*Figure 2A*). Importantly despite the relatively lower sensitivity, patients with asymmetric wall thickening on echocardiography again demonstrated troponin and BNP concentrations that were more than double the values in patients with concentric wall thickening (troponin I 13.5 [8.0,32.5] vs. 5.3 [3.6,11.2] ng/L, *P* = 0.001; and BNP 64.7 [28.1,130.5] vs. 24.3 [10.2,52.9] pg/mL, *P* < 0.001) (see Supplementary data online, *Table S5*).

### Clinical outcomes

**Figure 4:**
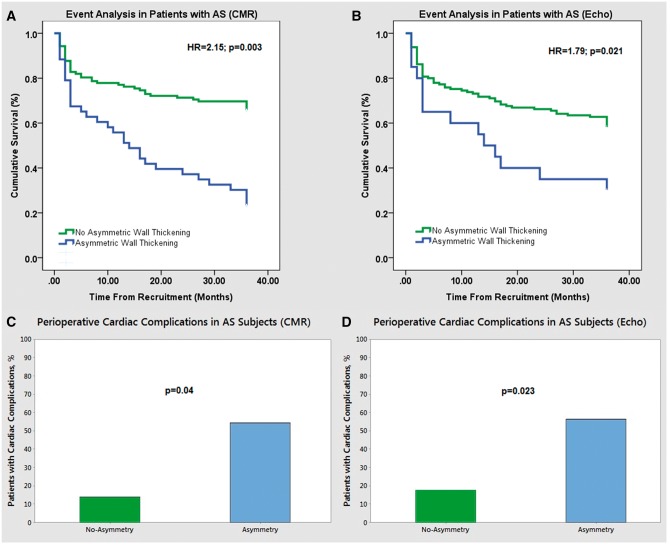
Outcome data in aortic stenosis patients with and without asymmetric wall thickening. Kaplan–Meier event estimates by adaptation patterns for the occurrence of death and AVR in aortic stenosis patients. Asymmetric thickening was associated with worse cardiac outcomes both when detected using magnetic resonance (*A*) (HR = 2.15 (1.29–3.59); *P* = 0.003) and echocardiography (*B*) (HR = 1.79 (1.08–3.69); *P* = 0.021). Perioperative complications in aortic stenosis patients undergoing aortic valve replacement. Subjects with asymmetric wall thickening had more cardiac complications in the perioperative period than those without based upon both magnetic resonance (*C*) 55% vs. 13% (*P* = 0.004) and echocardiographic (*D*) 57% vs. 19% (*P* = 0.023) assessments.

Patients were followed up for a median of 28 [22,33] months and 86 events occurred (72 aortic valve replacements and 14 patients died). Using the magnetic resonance analysis, the primary end-point was higher in patients with asymmetric wall thickening (*n* = 35, 81%) compared to both patients with concentric patterns (*n* = 34, 51%) and to all patients who did not have asymmetric wall thickening (*n* = 51, 42%). Indeed asymmetric wall thickening was associated with worse outcomes independent of age, sex, left ventricular mass index, coronary artery disease, and importantly aortic stenosis severity assessed with the mean aortic valve gradient [hazard ratio (HR) = 2.15; 95% confidence interval (CI) 1.29–3.59; *P* = 0.003](*Figure 4*). Among patients that underwent aortic valve replacement, perioperative complications were also more frequently observed in individuals with asymmetric wall thickening than subjects without (55% vs. 13%; *P* = 0.004; *Figure 4*).

Based upon the echocardiographic assessments, there were 23 outcome events (20 underwent aortic valve replacement and 3 died) in the 29 patients (79%) with asymmetrical wall thickening. This compared with 48 events in the 105 patients (46%) with concentric wall thickening. Using our combined primary outcome measure the asymmetric group on echo again had worse outcomes independent of age, sex, left ventricular mass index, coronary artery disease and the mean aortic valve gradient (HR = 1.79; 95% CI 1.08–3.69; *P* = 0.021). In patients that underwent aortic valve replacement, perioperative adverse outcomes were also more frequently reported in subjects with asymmetric wall thickening on echocardiographic (57% vs. 19%; *P* = 0.023).

## Discussion

We here provide a comprehensive multimodality imaging assessment of asymmetric wall thickening in 166 patients with aortic stenosis. In a prospective consecutive cohort, we demonstrate that asymmetric wall thickening is common, affecting a quarter (*n* = 43) of patients with mild-to-severe aortic stenosis when assessed using magnetic resonance. Echocardiography is less sensitive missing a third of these cases so that asymmetric wall thickening was only identified in 17% (*n* = 29). Irrespective of the imaging modality used, patients with asymmetric wall thickening had evidence of more advanced left ventricular decompenzation with elevated myocardial injury and increased BNP concentrations compared to those with concentric wall thickening. This was despite the two groups having similar co-morbidities, valve narrowing, myocardial fibrosis, and left ventricular hypertrophy. Moreover patients with asymmetric wall thickening (on both magnetic resonance and echocardiography) were found to have an adverse prognosis, with this form of remodelling acting as an independent predictor of aortic valve replacement or death after correction for age, sex, left ventricular mass index, coronary artery disease, and aortic stenosis severity.

Magnetic resonance has emerged as the gold standard non-invasive assessment of left ventricular mass and wall thickness. This is the first study to evaluate the true prevalence of asymmetric wall thickening in a cohort of patients with aortic stenosis, free from referral bias. This is in contrast to the only previous magnetic resonance study examining this question where patients were referred for magnetic resonance on clinical grounds. Unusual patterns of remodelling could therefore have potentially triggered the referral. Our data have demonstrated that asymmetric wall thickening is indeed common amongst patients with aortic stenosis affecting approximately a quarter of subjects (40% in those with severe stenosis), and characterized by advanced wall thickness measurements (16 [14,17] mm).

Echocardiography is less well suited to measuring wall thickness and limited by the availability of acoustic windows. In this study, it missed 14 cases of asymmetric wall thickening detected by magnetic resonance. In eight patients, this was because short-axis echocardiographic images were not interpretable. Overall our prevalence of asymmetric wall thickening on echocardiography is similar to that previously reported^7^^,^^8^ and to a recent analysis of the Intensive Lipid Lowering with Simvastatin and Ezetimibe in AS SEAS trial.^5^ Importantly patients with asymmetric wall thickening on echocardiography demonstrated the same characteristics as those detected by magnetic resonance including the increased troponin and BNP levels as well as the adverse prognosis (see Supplementary data online, *Table S3*). This is an important observation because echocardiography, unlike magnetic resonance, is performed routinely to assess all patients with aortic stenosis. It suggests that the presence of asymmetric wall thickening should therefore be actively looked for on echocardiography (not simply ignored and attributed to a ‘sigmoid septum’) and used to help identify patients likely to require aortic valve replacement rapidly and who might therefore benefit from more regular clinical follow-up.

Why do some patients develop asymmetric rather than concentric wall thickening in response to increased afterload? The explanation for this observation remains unclear. In this comprehensive evaluation, we did not observe any clear differences in patient demographics, co-morbidity, bicuspid aortic valve prevalence, or aortic stenosis severity between these two groups. Asymmetric wall thickening did appear reversible following surgery suggesting that it represents an adaptive response to an increased afterload. One potential theory relates to the bending radius of the septum and posterior wall of the LV. It has been suggested that the larger radius of the septum leads to greater myocardial tensions during contraction, promoting a more pronounced hypertrophic response.^12^^,^^13^ While this concept explains why asymmetric wall thickening is almost universally confined to the septum, it fails to account for inter-individual differences in the degree of asymmetric wall thickening. It is possible that patients may have a subtle genetic predisposition to regional wall thickening (similar to those driving the development of hypertrophic cardiomyopathy) that becomes clinically apparent with exposure to an increased left ventricular afterload.^14^ Unfortunately we do not have genetic data in this study to test this hypothesis. The mechanism for the adverse event rate in patients with asymmetric wall thickening also remains unclear, although it was predominantly driven by increased rates of aortic valve replacement. Asymmetric wall thickening was associated with evidence of increased myocardial injury (cTnI) and wall stress (BNP) suggesting that it is a marker of more advanced decompenzation and that patients will be more likely to develop symptoms and progress towards aortic valve replacement.^15^ The increased wall thickening may predispose patients to supply-demand ischaemia, increased myocyte injury and troponin levels, though this hypothesis requires confirmation. It is interesting that patients with asymmetric wall thickening did not have evidence of increased myocardial fibrosis, another useful marker of left ventricular decompenzation.

### Limitations

During follow-up, a total of 14 patients died, limiting our mortality assessments, the outcome data are therefore predominantly driven by aortic valve replacement. A large multicentre study with longer follow-up is desirable. Further attention should also be paid to the mechanism underlying asymmetric wall thickening formation, including the underlying genetics and the explanation for the associated adverse prognosis.

## Conclusions

Asymmetric wall thickening is a common adaptation pattern in patients with aortic stenosis, which can be detected using both cardiovascular magnetic resonance and echocardiography. Despite similar demographics, comorbidities, valve narrowing, myocardial hypertrophy, and fibrosis patients with asymmetric wall thickening have increased evidence of myocardial injury (with elevated cardiac troponin I) and BNP levels. Moreover, asymmetric wall thickening was associated with adverse outcomes acting as an independent predictor of aortic valve replacement or death in this population.

## Supplementary data

Supplementary data are available at *European Heart Journal—Cardiovascular Imaging* online.

## Supplementary Material

Supplementary Tables and Figures
