# Supplementary material for: Adverse prognosis associated with asymmetric myocardial thickening in aortic stenosis
Source: Eur Heart J Cardiovasc Imaging. 2017 Mar 30;19(3):347–56. doi: 10.1093/ehjci/jex052 (PMC5837366; doi:10.1093/ehjci/jex052)
Supplement: Supplementary Tables and Figures [file supplementary_data_jex052.pdf]

Adverse Prognosis Associated with Asymmetric Myocardial Thickening in Aortic Stenosis

Supplementary Data

Content:

1. Methods
   - Blood sampling and analysis.
   - Table S1. Magnetic resonance and echocardiographic definitions of six patterns of left ventricular adaption in aortic stenosis.

2. Results
   - Study Population Characteristics
   - Left Ventricular Characteristics of Patients with Aortic Stenosis on CMR
   - Left Ventricular Remodelling and Hypertrophy on CMR
   - Table S2. Baseline characteristics of patients with different forms of remodelling and hypertrophy on magnetic resonance.
   - Table S3. Comparison of patient characteristics between subjects with left ventricular remodelling and left ventricular hypertrophy.
   - Table S4. Comparison of patient characteristics between subjects with asymmetric wall thickening on echocardiography and magnetic resonance.
   - Table S5. Baseline characteristics of patients with different forms of remodelling and hypertrophy on Echocardiography.
   - Table S6. Comparison on patient characteristics between asymmetric and concentric wall thickening diagnosed using echocardiography.
   - Table S7. Hazard ratios predicting adverse events (aortic valve replacement or deaths) for asymmetric wall thickening on magnetic resonance and echocardiography in adjusted and unadjusted analyses.
   - Table S8. Hazard ratios predicting adverse events (aortic valve replacement or deaths) for variables, which were used to construct the models, presented in Table S7 in unadjusted analysis.
   - Table S8. Perioperative Cardiac Complication in AS subjects.

3. Figures
   - Figure S1. Further examples of asymmetric wall thickening on echocardiography and magnetic resonance imaging.

4. References

METHODS

Blood sampling and analysis

Plasma cardiac troponin I (cTnI) concentrations were measured using the Abbott Architect Stat high-sensitivity cTnI assay (Abbott Laboratories, Abbott Park, Il, USA). The lower limit of detection of this assay is 1.2 ng/L whilst the 99th centile from the reference population is 16ng/L for females and 34ng/L for males. We have previously shown that the analytical variability of the Architect Stat assay is low with a coefficient of variation for duplicate samples of 10% at 6 ng/L.[1] Brain natriuretic peptide (BNP) concentrations were measured using the Triage BNP assay (Biosite, Inc., San Diego, CA, USA). The inter-assay coefficient of variation was 10% at 28.8 pg/mL, with a detection range of 5–1300 pg/mL.[2]

Table S1. Magnetic resonance and echocardiographic definitions of six patterns of left ventricular adaption in aortic stenosis.

Asymmetric wall thickening on both modalities was defined by a maximal wall thickness ≥13 mm that was also ≥1.5-fold the thickness of the opposing myocardial segment.

		**Concentric Wall Thickening**		**Asymmetric Wall Thickening**		
	Normal Ventricle	Concentric remodelling	Concentric hypertrophy	Asymmetric remodelling	Asymmetric hypertrophy	Eccentric hypertrophy
**MAGNETIC RESONANCE**						
LV mass index, above age & gender normal values	-	-	✓	-	✓	✓
LV Mass/EDV ≥1.16 g/mL	-	✓	✓	✓	✓	-
Asymmetric wall thickening	-	-	-	✓	✓	-
LVEDVi above age & gender normal values	-	-	-	-	-	✓
**ECHOCARDIOGRAPHY**						
LV mass index, ≤95 g/m^2 for females ≤115 g/m^2 for males		-	✓	-	✓	✓
Relative wall thickness >0.42	-	✓	✓	✓	✓	-
Asymmetric wall thickening	-	-	-	✓	✓	-

LVEDVi, left ventricular end diastolic volume indexed to body surface; LV, left ventricular;

RESULTS

Study Population Characteristics

In the patient group, 34 (20%) had mild, 45 (27%) moderate, and 87 (53%) severe aortic stenosis. The mean peak aortic jet velocity and aortic valve area were 3.8±0.9 m/s and 1.0±0.4cm^2 respectively. Among patients with aortic stenosis, 4 (2%) had an ejection fraction <50% (45±2%). Overall 112 (67%) patients had a history of hypertension (mean systolic blood pressure 151±21 mmHg), 25 (15%) had diabetes mellitus and 59 (35%) had coronary artery disease (Table S2).

Left Ventricular Characteristics of Patients with Aortic Stenosis

Compared to controls, patients with aortic stenosis had increased maximum wall thickness measurements (7 [7,9] vs. 11 [9,14] mm; p<0.001), increased M/V (0.92 [0.84,0.98] *vs.* 1.24 [1.04,1.44] g/mL; p<0.001) and increased indexed left ventricular mass (62 [54,71] *vs.* 88 [73,99] g/m^2; p<0.001). None of the healthy volunteers had hypertrophy. There were no differences in left ventricular mass index among patients with aortic stenosis in the presence or absence of co-morbidities such as diabetes mellitus (p=0.42), hypertension (p=0.66), hyperlipidaemia (p=0.88) and coronary artery disease (p=0.24). Mean and maximum wall thickness measurements as well as the M/V ratio were also unrelated to the aforementioned co-morbidities (all p>0.20).

Left Ventricular Remodelling and Hypertrophy

Compared to patients exhibiting concentric and asymmetric left ventricular hypertrophy (eccentric forms were excluded due to the other factors driving remodelling), subjects with left ventricular remodelling had lower peak aortic jet velocities (3.9 [3.3,4.3] vs. 4.2 [3.7,4.8] m/s; p=0.008) with an observed tendency to larger aortic valve areas (0.9 [0.8,1.1] cm^2 *vs.* 0.8 [0.7,1.0]; p=0.07) although differences were small. Despite no differences in systolic function (left ventricular ejection fraction and longitudinal function both p>0.2), diastolic function was impaired (E/e' 14.2 [11.5,19.5] and 11.6 [9.6,13.9], p=0.0041) and myocardial

fibrosis higher (53% vs 18% with mid-wall LGE, $p < 0.001$; ECV 28.4±2.9 *vs*. 26.4±1.8%, $p<0.001$) in patients with hypertrophy versus those with remodelling (Table S2).

Table S2. Baseline characteristics of patients with different forms of remodelling and hypertrophy on magnetic resonance

	**Normal ventricle (n=39)**	**Concentric remodelling (n=22)**	**Asymmetric remodelling (n=12)**	**Concentric hypertrophy (n=45)**	**Asymmetric hypertrophy (n=31)**	**Eccentric hypertrophy (n=17)**	**P Value**
**BASELINE CHARACTERISTICS**							
Age, years	69 [55, 75]	71 [66, 76]	72 [67, 73]	70 [64, 78]	72 [66, 77]	68 [65, 77]	0.40
Males, n (%)	21 (58)	20 (91)	10 (83)	32 (71)	21 (68)	9 (56)	0.06
CAD, n (%)	12 (33)	7 (32)	5 (42)	15 (33)	15 (48)	4 (21)	0.49
Diabetes, n (%)	5 (14)	5 (23)	0 (0)	4 (9)	7 (23)	3 (16)	0.31
Hyperlipidaemia, n (%)	13 (37)	13 (59)	8 (67)	20 (44)	14 (45)	7 (39)	0.34
Hypertension, n (%)	20 (56)	17 (77)	9 (75)	31 (69)	24 (77)	11 (58)	0.22
SBP, mmHg	147±23	149±18	148±20	151±22	155±22	154±19	0.71
Six minute walk, distance (m)	424±73	368±63	380±90	370±111	345±135	389±50	0.04
Symptomatic AS, n (%)	4 (10)	6 (27)	3 (25)	10 (22)	11 (35)	3 (18)	0.26
**ECHOCARDIOGRAPHY**							
Vm, m/s	3.0 [2.5, 3.8]	3.5 [2.9, 3.9]	4.1 [3.9, 4.6]	4.1 [3.5, 4.6]	4.2 [3.8, 4.9]	3.7 [3.2, 4.0]	<0.001
MPG, mmHg	20 [14, 32]	28 [17, 37]	40 [36, 46]	36 [28, 48]	41 [31, 54]	27 [22, 38]	<0.001
- Mild, n (%)	19 (49)	5 (23)	1 (8)	5 (11)	0	0	<0.001
- Moderate, n (%)	9 (23)	8 (36)	2 (17)	10 (22)	7 (23)	9 (53)	0.265
- Severe, n (%)	8 (21)	9 (41)	9 (75)	30 (67)	24 (77)	8 (47)	<0.001
AVA, cm^2	1.0 [0.8, 1.3]	1.0 [0.8, 1.2]	0.8 [0.7, 1.0]	0.8 [0.7, 1.0]	0.8 [0.7, 1.0]	1.0 [0.8, 1.1]	0.002
AVA indexed, cm^2/m^2	0.55 [0.45, 0.71]	0.52 [0.41, 0.63]	0.45 [0.35, 0.49]	0.41 [0.37, 0.49]	0.42 [0.34, 0.51]	0.50 [0.43, 0.59]	0.001
Dimensionless index	0.32 [0.25, 0.41]	0.28 [0.24, 0.32]	0.23 [0.19, 0.25]	0.24 [0.19, 0.30]	0.24 [0.19, 0.29]	0.25 [0.23, 0.32]	<0.001
Indexed SV <35mL/m^2, n (%)	8 (22)	7 (31)	3 (25)	6 (14)	6 (19)	3 (16)	0.60
E/A	0.96 [0.74, 1.24]	0.77 [0.69, 1.05]	0.71 [0.52, 0.97]	0.84 [0.70, 1.00]	0.89 [0.65, 1.16]	1.11 [0.80, 1.25]	0.013
Deceleration Time, ms	188 [167, 225]	206 [163,277]	217 [150,246]	198 [171, 250]	227 [187, 248]	186 [166, 231]	0.050
E/e'	11.4 [8.9, 13.7]	11.0 [9.0, 12.8]	12.4 [10.4, 16.9]	13.9 [11.0, 19.0]	15.1 [11.8, 22.5]	12.4 [11.2, 16.0]	0.005
LVOT Vm, m/s	1.0 [0.9, 1.1]	1.0 [0.9, 1.1]	0.9 [0.9, 1.1]	1.0 [0.9, 1.2]	1.0 [0.9, 1.2]	0.9 [0.9, 1.2]	0.82
Bicuspid Aortic Valve n (%)	13 (36)	8 (36)	3 (25)	18 (40)	11 (35)	4 (21)	0.75
**CARDIOVASCULAR MAGNETIC RESONANCE**							
Indexed EDV, mL/m^2	68 [60, 77]	61 [50, 68]	61 [50, 67]	69 [62, 78]	75 [64, 84]	88 [71, 114]	<0.001
Indexed ESV, mL/m^2	24 [20, 27]	17 [13, 23]	18 [14, 22]	22 [18, 27]	24 [21, 27]	26 [24,.40]	<0.001
Indexed SV, mL/m^2	45±8	42±7	42±7	47±10	50±9	59±12	<0.001
Indexed SV <35mL/m^2, n (%)	2 (6%)	2 (9%)	2 (16%)	4 (9%)	2 (6%)	0 (0%)	NA
Ejection fraction, %	66 [62,.68]	70 [64, 73]	69 [66, 75]	67 [63, 71]	67 [64, 72]	65 [62, 69]	0.08
Longitudinal function, mm	13.5±2.6	13.0±2.6	11.0±2.5	11.1±3.0	11.1±2.6	13.6±2.8	<0.001
Max Wall Thickness, mm	8 [6, 9]	10 [9, 12]	15 [14,16]	12 [11, 14]	17 [15, 18]	10 [8, 12]	<0.001
Indexed left ventricular mass, g/m^2	64 [59, 77]	80 [73, 87]	78 [75, 83]	98 [92, 108]	101 [93, 114]	96 [78, 104]	<0.001
Left ventricular mass/EDV, g/mL	0.97 [0.89, 1.05]	1.27 [1.22, 1.46]	1.30 [1.22, 1.48]	1.42 [1.28, 1.62]	1.41 [1.20, 1.51]	1.03.[0.93, 1.07]	<0.001
Mid-wall Fibrosis, n (%)	5 (14)	4 (18)	2 (17)	21 (47)	19 (61)	8 (50)	<0.001
Extracellular volume fraction, %	27.2±2.0	26.5±1.8	26.4±1.7	28.1±3.1	28.8±2.5	28.4±3.0	0.003
Indexed Fibrosis volume, mL/m^2	17.5 [16.3, 20.0]	20.8 [18.4, 23.6]	20.0 [18.9, 22.5]	27.2 [23.9, 31.8]	28.1 [26.1, 32.3]	23.6 [21.0, 28.7]	<0.001
**BIOMARKERS**							
HS-cTnI, ng/L	3.1 [1.5, 4.3]	5.3 [3.7, 7.4]	13.5 [7.4, 33.0]	8.0 [4.4, 15.2]	13.8 [8.2, 28.0]	6.3 [3.3, 9.9]	<0.001
BNP, pg/mL	21.9 [8.3, 40.1]	13.6 [5.9, 33.9]	27.2 [14.0, 41.4]	21.6 [11.0, 54.1]	65.6[30.2,123.8]	27.5 [8.4, 56.3]	0.001
**OUTCOMES**							
Combined Primary Outcome, n (%)	6 (15)	11 (50)	10 (83)	23 (51)	25 (81)	6 (38)	<0.001

AVR, n (%)	6 (15)	9 (41)	9 (75)	19 (42)	22 (71)	6 (38)	<0.001
AS-related death, n (%)	0	1 (5)	0	1 (2)	2 (6)	0	NA
All-cause death, n (%)	0	2 (9)	1 (8)	5 (11)	5 (16)	1 (6)	0.09

CAD, coronary artery disease; SBP, systolic blood pressure; MPG, mean pressure gradient; LVOT, left ventricular outflow track; EDV, end diastolic volume; ESV, end systolic volume; SV, stroke volume; HS-cTnI, high-sensitivity cardiac troponin I; AVR, aortic valve replacement

Table S3. Comparison of patient characteristics between subjects with left ventricular remodelling and left ventricular hypertrophy on magnetic resonance. Patients with eccentric hypertrophy were excluded.

	**Left ventricular remodelling (n=34)**	**Left ventricular hypertrophy (n=76)**	**P Value**
**BASELINE CHARACTERISTICS**			
Age, years	71 [67, 75]	72 [65, 78]	0.78
Males, n (%)	30 (88)	53 (70)	0.03
CAD, n (%)	12 (35)	30 (39)	0.68
Diabetes, n (%)	5 (15)	11 (14)	0.97
Hyperlipidaemia, n (%)	21 (62)	34 (45)	0.10
Hypertension, n (%)	26 (76)	55 (72)	0.65
SBP, mmHg	148±19	152±21	0.33
Six minute walk, distance (m)	374±73	360±121	0.47
Symptomatic AS, n (%)	9 (26)	21 (27)	0.90
**ECHOCARDIOGRAPHY**			
AVA, cm^2	0.9 [0.8, 1.1]	0.8 [0.7, 1.0]	0.07
AVA indexed, cm^2/m^2	0.45 [0.40, 0.57]	0.42 [0.37, 0.51]	0.10
Dimensionless index	0.25 [0.23, 0.31]	0.24 [0.20, 0.30]	0.26
Vm, m/s	3.9 [2.5, 3.8]	4.2 [3.7, 4.8]	0.01
MPG, mmHg	35 [21.0, 42]	38 [29, 50]	0.01
- Mild, n (%)	6 (18)	5 (7)	0.09
- Moderate, n (%)	10 (29)	17 (22)	0.43
- Severe, n (%)	18 (53)	54 (71)	0.07
Indexed SV <35mL/m^2, n (%)	10 (29)	12 (16)	0.11
E/A	0.74 [0.65, 1.01]	0.85 [0.69, 1.10]	0.46
Deceleration Time, ms	211 [160, 256]	214 [178, 247]	0.67
E/e'	11.6 [9.6, 13.9]	14.2 [11.5, 19.5]	0.001
LVOT Vm, m/s	0.9 [0.9, 1.1]	1.0 [0.9, 1.1]	0.21
Bicuspid Aortic Valve n (%)	11 (32)	29 (38)	0.56
**CARDIOVASCULAR MAGNETIC RESONANCE**			
Indexed EDV, mL/m^2	61 [50, 67]	72 [63, 80]	<0.001
Indexed ESV, mL/m^2	17 [14, 23]	23 [19, 27]	<0.001
Indexed SV, mL/m^2	42±6	48±9	0.001
Indexed SV <35mL/m^2, n (%)	4 (11%)	6 (8%)	0.52
Ejection fraction, %	70 [64, 74]	67 [63, 72]	0.036
Longitudinal function, mm	12.3±2.7	11.1±2.8	0.046
Max Wall Thickness, mm	11 [9, 14]	14 [12, 16]	0.001
Indexed left ventricular mass, g/m^2	79 [73, 86]	99 [92, 110]	<0.001
Left ventricular mass/EDV, g/mL	1.28 [1.22, 1.45]	1.41 [1.26, 1.58]	0.017

Mid-wall Fibrosis, n (%)	6 (18)	40 (53)	<0.001
Extracellular volume fraction, %<br>Indexed Fibrosis volume, mL/m^2	26.4±1.8<br>20.3 [18.7, 23.2]	28.4±2.9<br>27.7 [24.4, 32.1]	<0.001<br><0.001
**BIOMARKERS**			
HS-cTnI, ng/L<br>BNP, pg/mL	6.9 [4.7, 11.6]<br>16.5 [7.9, 37.8]	9.5 [5.0, 18.5]<br>41.1 [15.0, 81.7]	0.12<br>0.01
**OUTCOMES**			
Combined Primary Outcome, n (%)<br>AVR, n (%)<br>AS-related death, n (%)<br>All cause death, n (%)	21 (62)<br>18 (53)<br>1 (3)<br>3 (9)	48 (63)<br>41 (54)<br>3 (4)<br>10 (13)	0.89<br>0.92<br>0.88<br>0.51

CAD, coronary artery disease; SBP, systolic blood pressure; MPG, mean pressure gradient; LVOT, left ventricular outflow track; EDV, end diastolic volume; ESV, end systolic volume; SV, stroke volume; HS-cTnI, high-sensitivity cardiac troponin I; AVR, aortic valve replacement

Table S4. Comparison of patient characteristics between subjects with asymmetric wall thickening on echocardiography and cardiovascular magnetic resonance imaging.

	**Asymmetric Wall Thickening (Echocardiography) (n=29)**	**Asymmetric Wall Thickening (magnetic resonance) (n=43)**	**P Value**
**BASELINE CHARACTERISTICS**			
Age, years	73 [69, 81]	72 [67, 75]	0.39
Males, n (%)	19 (65)	31 (72)	0.58
CAD, n (%)	17 (58)	20 (47)	0.33
Diabetes, n (%)	5 (17)	7 (16)	0.87
Hyperlipidaemia, n(%)	17 (58)	22 (51)	0.58
Hypertension, n(%)	23 (80)	33 (77)	0.74
SBP, mmHg	152±21	153±22	0.91
Six minute walk, distance.(m)	365±127	358±124	0.92
Symptomatic AS, n(%)	13 (45)	16 (37)	0.52
**ECHOCARDIOGRAPHY**			
AVA, cm^2	0.8 [0.6, 1.0]	0.8 [0.7, 1.0]	0.77
AVA indexed, cm^2/m^2	0.45 [0.32, 0.51]	0.43 [0.36, 0.50]	0.91
Dimensionless index	0.23 [0.19, 0.30]	0.23 [0.19, 0.28]	0.49
Vm, m/s	4.4 [4.0, 5.1]	4.2 [3.9, 4.9]	0.60
MPG, mmHg	45 [35, 60]	41 [35, 50]	0.54
- Mild, n (%)	0	1 (2)	NA
- Moderate, n (%)	4 (14)	9 (21)	0.43
- Severe, n (%)	25 (86)	33 (77)	0.32

Indexed SV <35mL/m^2, n(%)	6 (21)	9 (21)	0.87
E/A	0.79 [0.61, 1.12]	0.82 [0.63, 1.16]	0.65
Deceleration Time	223 [200, 247]	217 [196, 247]	0.73
E/e'	16.8 [12.8, 24.4]	14.2 [11.5, 18.5]	0.27
LVOT Vm, m/s	1.0 [0.9, 1.2]	1.0 [0.9, 1.2]	0.60
Bicuspid Aortic Valve n(%)	7 (24)	14 (33)	0.59
**CARDIOVASCULAR MAGNETIC RESONANCE**			
Indexed EDV, mL/m^2	71 [64, 80]	68 [62, 78]	0.40
Indexed ESV, mL/m^2	22 [18, 25]	22 [18, 26]	0.80
Indexed SV, mL/m^2	50±10	48±9	0.32
Indexed SV <35mL/m^2, n(%)	2 (7%)	4 (8%)	0.94
Ejection fraction, %	68 [65, 72]	67 [64, 73]	0.78
Longitudinal function, mm	10.9±2.5	11.1±2.6	0.84
Max Wall Thickness, mm	16 [15, 18]	16 [14, 17]	0.55
Indexed left ventricular mass, g/m^2	101 [89, 113]	96 [80, 106]	0.32
Left ventricular mass/EDV, g/mL	1.37 [1.24, 1.56]	1.36 [1.21, 1.50]	0.63
Mid-wall Fibrosis, n(%)	13 (44)	21 (48)	0.44
Extracellular volume fraction, %	28.7±2.7	28.1±2.6	0.46
Indexed Fibrosis volume, mL/m^2	24.9 [20.4, 28.7]	26.6 [21.1, 30.4]	0.19
**BIOMARKERS**			
HS-cTnI, ng/L	13.5 [8.0, 32.5]	13.5 [8.1, 32.8]	0.71
BNP, pg/mL	64.7.[28.1, 130.5]	56.3 [25.5, 112]	0.24
**OUTCOMES**			
Combined Primary Outcome, n (%)	23 (79)	35 (81)	0.55
AVR, n(%)	20 (69)	31 (72)	0.53
AS-related death, n(%)	1 (4)	2 (5)	NA
All cause death, n(%)	3 (10)	6 (14)	0.74

CAD, coronary artery disease; SBP, systolic blood pressure; MPG, mean pressure gradient; LVOT, left ventricular outflow track; EDV, end diastolic volume; ESV, end systolic volume; SV, stroke volume; HS-cTnI, high-sensitivity cardiac troponin I; AVR, aortic valve replacement

Table S5. Baseline characteristics of patients with different forms of remodelling and hypertrophy on echocardiography.

	Normal ventricle (n=25)	Concentric remodelling (n=37)	Asymmetric remodelling (n=4)	Concentric hypertrophy (n=68)	Asymmetric hypertrophy (n=25)	Eccentric hypertrophy (n=8)	P Value
**BASELINE CHARACTERISTICS**							
Age, years	67 [62, 73]	70 [65, 77]	73 [71, 83]	70 [63, 75]	72 [67, 78]	74 [67, 78]	0.18
Males, n (%)	13 (52)	23 (62)	4 (100)	52 (76)	17 (68)	5 (63)	0.12
CAD, n (%)	4 (16)	12 (32)	2 (50)	22 (32)	12 (48)	3 (38)	0.07
Diabetes, n (%)	4 (16)	4 (11)	0 (0)	10 (15)	4 (16)	2 (25)	NA
Hyperlipidaemia, n (%)	8 (32)	16 (43)	4 (100)	32 (47)	13 (52)	2 (25)	0.24
Hypertension, n (%)	15 (60)	21 (78)	3 (75)	42 (62)	22 (88)	8 (100)	0.04
SBP, mmHg	150±24	151±22	147±19	149±20	154±21	155±24	0.47
Six minute walk, distance (m)	397±89	375±132	375±45	378±92	373±119	384±71	0.05
**ECHOCARDIOGRAPHY**							
Vm, m/s	3.6 [2.9, 4.1]	3.5 [2.8, 3.9]	3.9 [3.4, 4.0]	4.0 [3.3, 4.5]	4.5 [4.0, 4.8]	3.8 [3.1, 4.2]	<0.001
MPG, mmHg	25 [16, 36]	25 [16, 37]	36 [26, 41]	38 [26, 44]	44 [35, 57]	35 [16, 41]	<0.001
AVA, cm^2	1.0 [0.8, 1.3]	1.0 [0.8, 1.2]	1.0 [0.8, 1.1]	0.8 [0.7, 1.1]	0.8 [0.7, 1.0]	1.0 [0.8, 1.1]	0.087
AVA indexed, cm^2/m^2	0.53 [0.45, 0.68]	0.53 [0.40, 0.70]	0.51 [0.35, 0.62]	0.46 [0.38, 0.58]	0.45 [0.34, 0.49]	0.50 [0.43, 0.64]	0.042
Dimensionless index	0.29 [0.25, 0.38]	0.27 [0.24,0.34]	0.23 [0.19, 0.25]	0.24 [0.19, 0.30]	0.24 [0.19, 0.29]	0.25 [0.23, 0.32]	0.001
Indexed SV <35mL/m^2, n (%)	6 (24)	9 (24)	1 (25)	14 (20)	3 (12)	0 (0)	0.38
E/A	1.01 [0.79, 1.26]	0.79 [0.69, 1.08]	0.73 [0.52, 0.99]	0.82 [0.70, 1.08]	0.85 [0.68, 1.14]	1.11 [0.80, 1.25]	0.063
Deceleration Time	188 [167, 225]	206 [163,277]	217 [150,246]	198 [171, 250]	227 [187, 248]	186 [166, 231]	0.15
E/e'	12.9 [10.3, 14.9]	11.6 [8.7, 13.8]	13.1 [10.3, 16.3]	12.6 [10.6, 18.0]	16.6 [12.1, 22.4]	12.3 [8.7, 19.8]	0.027
LVOT Vm, m/s	1.0 [0.9, 1.1]	1.0 [0.9,1.1]	1.0 [0.9, 1.1]	0.9 [0.8, 1.1]	0.9 [0.9, 1.2]	0.9 [0.9, 1.1]	0.82
Bicuspid Aortic Valve n (%)	9 (36)	8 (22)	1 (25)	28 (41)	8 (32)	3 (37)	0.46
**CARDIOVASCULAR MAGNETIC RESONANCE**							
Indexed EDV, mL/m^2	63 [59, 74]	64 [57, 71]	64 [52, 80]	74 [62, 86]	75 [67, 89]	89 [72, 114]	0.001
Indexed ESV, mL/m^2	21 [18, 25]	22 [17, 25]	21 [14, 24]	25 [21, 29]	22 [19, 28]	26 [24, 40]	0.02
Indexed SV, mL/m^2	45±8	43±7	45±10	50±11	51±9	59±12	0.004
Indexed SV <35mL/m^2, n (%)	2 (8%)	2 (5%)	1 (25%)	5 (7%)	2 (8%)	0 (0%)	NA
Ejection fraction, %	69 [65, 73]	67 [64, 70]	70 [65, 74]	66 [63, 71]	67 [64, 74]	64 [60, 69]	0.187
Longitudinal function, mm	13.3±3.4	13.0±2.5	10.9±3.3	12.2±3.0	10.9±2.3	10.8±1.8	0.002
Max Wall thickness (mm)	8 [6, 9]	10 [9, 12]	15 [14,16]	12 [11, 14]	17 [15, 18]	9 [8, 11]	<0.001
Indexed LV mass, g/m^2	69 [60, 77]	74 [68, 89]	84 [75, 99]	97 [91, 108]	101 [91, 115]	95 [83, 104]	<0.001
LV mass/EDV, g/mL	0.99 [0.92, 1.06]	1.22 [1.17, 1.32]	1.31 [1.20, 1.51]	1.36 [1.18, 1.53]	1.41 [1.19, 1.53]	1.03 [0.94, 1.09]	0.014
Mid-wall Fibrosis, n (%)	3 (12)	9 (33)	1 (25)	27 (40)	16 (64)	3 (38)	<0.001
Extracellular volume fraction, %	27.1±2.5	27.7±2.3	26.6±1.1	27.8±2.7	28.9±2.6	26.7±3.3	0.003
Indexed Fibrosis volume, mL/m^2	17.5 [16.7, 22.0]	20.1 [17.7, 24.6]	22.9 [19.0, 27.0]	26.0 [20.7, 30.9]	28.1 [25.7, 33.9]	23.8 [19.9, 27.4]	<0.001
**BIOMARKERS**							
HS-cTnI, ng/L	3.4 [1.7, 4.9]	4.5 [2.5, 9.1]	6.8 [3.9, 14.5]	7.0 [3.9, 11.4]	14.8 [9.6, 38.3]	6.7 [4.9, 11.0]	<0.001
BNP, pg/mL	17.8 [5.9, 30.2]	19.1 [7.0, 38.4]	29.4 [26.9, 43.2]	27.5 [14.8, 63.4]	72.9 [30.8, 141.8]	29.7 [16.4, 72.3]	0.001
**OUTCOMES**							
Combined Primary Outcome, n (%)	10 (40)	10 (27)	4 (100)	34 (50)	18 (72)	5 (62)	0.001
AVR, n (%)	9 (15)	8 (22)	4 (100)	30 (44)	15 (60)	5 (62)	0.005
AS-related death, n (%)	0	1 (3)	0	1 (1)	2 (8)	0	NA
All-cause death, n (%)	0	2 (5)	0	7 (10)	4 (16)	1 (12)	NA

CAD, coronary artery disease; SBP, systolic blood pressure; MPG, mean pressure gradient; LVOT, left ventricular outflow track; EDV, end diastolic volume; ESV, end systolic volume; SV, stroke volume; HS-cTnI, high-sensitivity cardiac troponin I; AVR, aortic valve replacement

Table S6. Comparison of patient characteristics between asymmetric and concentric wall thickening diagnosed using echocardiography. Patients with eccentric hypertrophy were excluded

	Asymmetric Wall Thickening (Echocardiography) (n=29)	Concentric Wall Thickening (Echocardiography) (n=105)	P Value
**BASELINE CHARACTERISTICS**			
Age, years	73 [69, 81]	70 [63, 74]	0.13
Males, n (%)	19 (65)	79 (71)	0.58
CAD, n (%)	17 (58)	37 (33)	0.03
DM, n (%)	5 (17)	18 (16)	0.89
Hyperlipidaemia, n (%)	17 (58)	48 (43)	0.16
Hypertension, n (%)	23 (80)	72 (65)	0.17
SBP, mmHg	152±21	151±21	0.76
Six minute walk, distance (m)	365±127	381±100	0.60
**ECHOCARDIOGRAPHY**			
AVA, cm^2	0.8 [0.6, 1.0]	0.9 [0.7, 1.2]	0.050
AVA indexed, cm^{2}/m^2	0.45 [0.32, 0.51]	0.44 [0.36, 0.56]	0.28
Dimensionless index	0.23 [0.19, 0.30]	0.26 [0.23, 0.32]	0.049
Vm, m/s	4.4 [3.9, 5.1]	3.8 [3.2, 4.3]	0.001
MPG, mmHg	45 [35, 60]	32 [22, 41]	0.003
Indexed SV <35mL/m^2, n (%)	6 (21)	19 (18)	0.75
E/A	0.79 [0.61, 1.12]	0.87 [0.65, 1.10]	0.68
Deceleration Time	223 [200, 247]	207 [183, 247]	0.41
E/e'	16.8 [12.8, 24.4]	12.4 [9.8, 16.6]	0.023
LVOT Vm, m/s	1.0 [0.9,1.2]	1.0 [0.9, 1.2]	0.21
Bicuspid Aortic Valve n (%)	7 (24)	38 (35)	0.41
**CARDIOVASCULAR MAGNETIC RESONANCE**			
Indexed EDV, mL/m^2	71 [64, 80]	68 [61, 79]	0.50
Indexed ESV, mL/m^2	22 [18, 25]	23 [18, 27]	0.89
Indexed SV, mL/m^2	50±10	51±9	0.26
Indexed SV <35mL/m^2, n (%)	2 (7)	10 (9)	0.89
Ejection fraction, %	68 [65, 72]	67 [63, 71]	0.51
Longitudinal function, mm	10.9±2.5	12.5±2.9	0.02
Indexed LV mass, g/m^2	101 [89, 113]	96 [85, 107]	0.005
LV mass/EDV, g/mL	1.37 [1.24, 1.56]	1.23 [1.04, 1.43]	0.003
Mid-wall Fibrosis, n (%)	13 (44)	36 (32)	0.021
Extracellular volume fraction, %	28.7±2.7	27.7±2.6	0.13
Indexed Fibrosis volume, mL/m^2	24.9. [20.4, 28.7]	24.4 [20.1, 28.4]	0.48
**BIOMARKERS**			
HS-cTnI, ng/L	13.5 [8.0, 32.5]	5.35 [3.6, 11.2]	0.001
BNP, pg/mL	64.7.7 [28.1, 130.5]	24.3 [10.2, 52.9]	<0.001
**OUTCOMES**			
Combined Primary Outcome, n (%)	23 (79)	48 (43)	0.008
AVR, n (%)	20 (69)	42 (38)	0.024
Cardiac death, n (%)	1 (4)	2 (2)	NA
All cause death, n (%)	3 (10)	7 (6)	0.21

CAD, coronary artery disease; SBP, systolic blood pressure; MPG, mean pressure gradient; LVOT, left ventricular outflow track; EDV, end diastolic volume; ESV, end systolic volume; SV, stroke volume; HS-cTnI, high-sensitivity cardiac troponin I; AVR, aortic valve replacement

Table S7. Hazard ratios predicting adverse events (aortic valve replacement or deaths) for asymmetric wall thickening on magnetic resonance and echocardiography in adjusted and unadjusted analyses.

	Asymmetric Wall Thickening on Magnetic Resonance		Asymmetric Wall Thickening on Echocardiography	
	Hazard ratio (95% CI)	p-value	Hazard ratio (95% CI)	p-value
Model 1	2.97 (1.87-4.69)	<0.001	2.28 (1.26-3.73)	0.008
Model 2	2.79 (1.73-4.59)	<0.001	2.08 (1.16-3.72)	0.014
Model 3	2.76 (1.69-4.52)	0.001	2.03 (1.11-3.70)	0.01
Model 4	2.21 (1.34-3.68)	0.004	1.86 (1.09-3.42)	0.014
Model 5	2.14 (1.28-3.57)	0.004	1.83 (1.10-3.53)	0.014
Model 6	2.15 (1.29-3.59)	0.003	1.79 (1.08-3.69)	0.021

Model 1 – unadjusted; Model 2 – adjusted for age and sex; Model 3 – similar to Model 2 additionally adjusted for indexed left ventricular mass; Model 4 – similar to Model 2 additionally adjusted for mean aortic valve pressure gradient; Model 5 – adjusted for age, sex, indexed left ventricular mass and mean aortic valve pressure gradient; Model 6 – similar to Model 5 additionally adjusted for Coronary Artery Disease

Table S8. Hazard ratios predicting adverse events (aortic valve replacement or deaths) for variables, which were used to construct the models, presented in Table S7 in unadjusted analysis.

Variable	Hazard ratio (95% CI)	p-value
Age	1.02 (1.00-1.05)	0.043
Gender	0.83 (0.51-1.33)	0.44
LV mass (indexed)	1.01 (0.99-1.02)	0.15
Mean Pressure Gradient	1.49 (1.23-1.82)	0.003
Coronary Artery Disease	1.15 (0.72-1.83)	0.56

Table S9. Perioperative Cardiac Complication in AS subjects.

Complication	Asymmetric Wall Thickening (n=35)	No Asymmetric Wall Thickening (n=37)	P value
Congestive Heart Failure, n (%)	5 (14)	1 (3)	NA
Atrial Fibrillation, n (%)	6 (17)	2 (5)	NA
Arrhythmia requiring Permanent Pacemaker Implantation, n (%)	5 (14)	2 (3)	NA
Perivalvular Leaks, n (%)	2 (6)	0	NA
Cardiac Tamponade, n (%)	1 (3)	0	NA
All Complications, n (%)	19 (55)	5 (13)	0.004

Figure S1. Further examples of asymmetric wall thickening on magnetic resonance (A-F) and echocardiography (G-I).

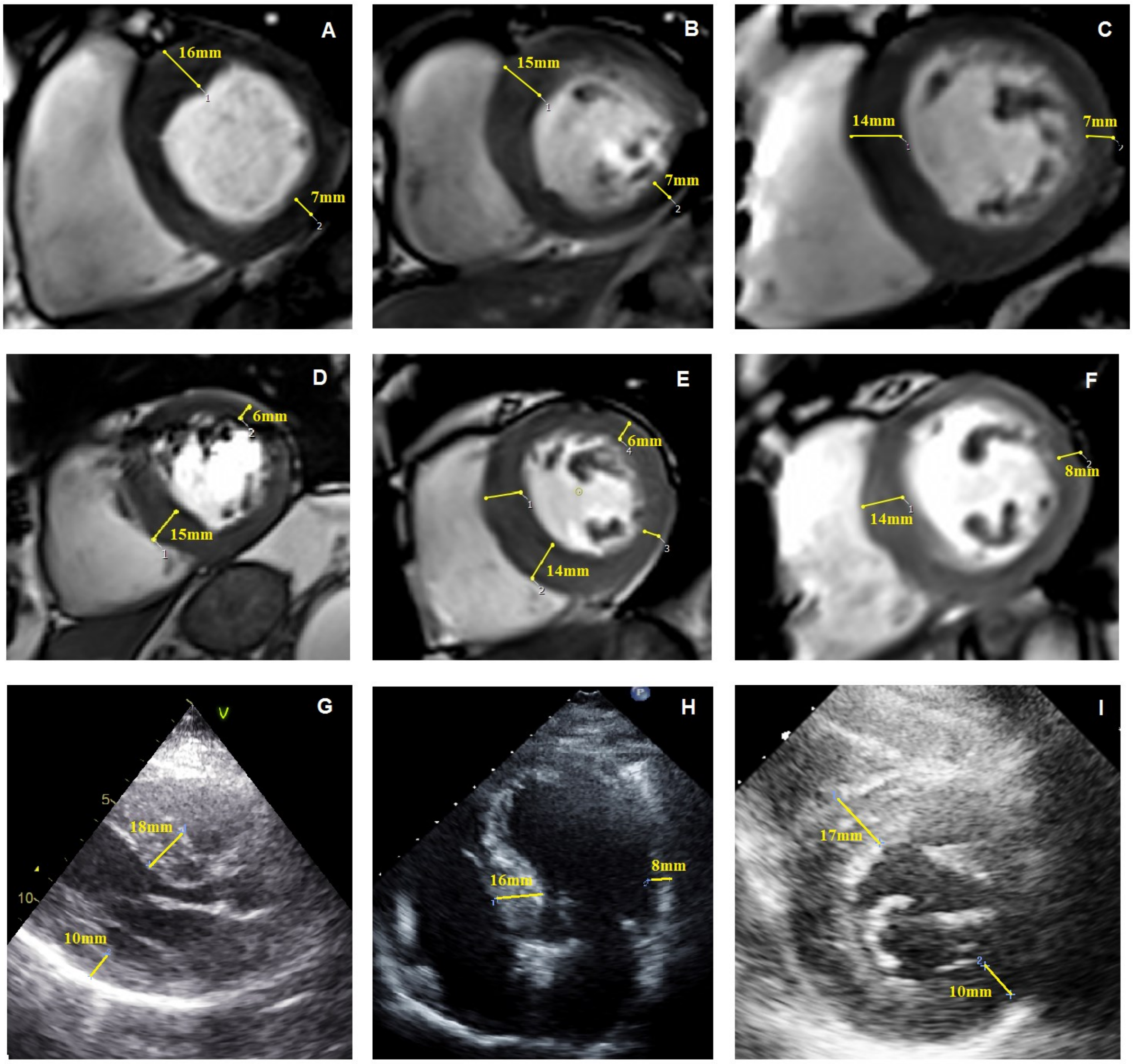

REFERENCES

1. Chin CWL, Shah ASV, McAllister DA, Cowell SJ, Alam S, Langrish JP, Strachan FE, Hunter AL, Choy AM, Lang CC, Walker S, Boon NA, Newby DE, Mills NL, Dweck MR. High-sensitivity troponin I concentrations are a marker of an advanced hypertrophic response and adverse outcomes in patients with aortic stenosis. European Heart Journal 2014;**35**(34):2312-2321.
2. Wieczorek SJ, Wu AHB, Christenson R, Krishnaswamy P, Gottlieb S, Rosano T, Hager D, Gardetto N, Chiu A, Bailly KR, Maisel A. A rapid B-type natriuretic peptide assay accurately diagnoses left ventricular dysfunction and heart failure: A multicenter evaluation. American Heart Journal 2002;**144**(5):834-839.
